# Nonconventional Tether Structure for Quality Factor Enhancement of Thin-Film-Piezoelectric-on-Si MEMS Resonator

**DOI:** 10.3390/mi14101965

**Published:** 2023-10-22

**Authors:** Mohammed Awad, Temesgen Bailie Workie, Jingfu Bao, Ken-ya Hashimoto

**Affiliations:** School of Integrated Circuits Science and Engineering, University of Electronic Science and Technology of China, Chengdu 611731, China; wtbailie@std.uestc.edu.cn (T.B.W.); k.hashimoto@ieee.org (K.-y.H.)

**Keywords:** MEMS, TPoS, anchor loss, quality factor, tether

## Abstract

This article presents a new design of supporting tethers through the concept of force distribution. The transmitted force applied on tethers will be distributed on the new tether design area, resulting in low acoustic energy transferred to anchor boundaries and stored energy enhancement. This technique achieves an anchor quality factor of 175,000 compared to 58,000 obtained from the conventional tether design, representing a three-fold enhancement. Furthermore, the unloaded quality factor of the proposed design improved from 23,750 to 27,442, representing a 1.2-fold improvement.

## 1. Introduction

Despite all performance enhancements to the IC industry and micro-fabrication, quartz crystal oscillators and surface acoustic wave devices are commonly used in transmitters and receivers of wireless architectures [1,2,3,4,5]. These components are widely spread in microelectronic markets due to their excellent properties [6,7,8]. One of these properties is low impedance characteristics, which are strongly preferred in constructing low insertion loss filters and low phase noise oscillators [9]. In addition to this, the quartz resonators have a high-quality factor that strongly supports their market’s acceptability. A high-resonator-quality factor reduces phase noise in oscillators [10,11] and enhances the filter selectivity [12,13]. 

These intrinsic qualities make decades of searching for other oscillating devices unsuccessful. The drawbacks of quartz oscillators are their large size and the impossibility of being fabricated with the electronic device at the die level [14]. In this regard, microelectromechanical systems (MEMS) strongly introduce themselves as a suitable substitutional to quartz oscillator [15,16]. Micromachined resonators are structures that vibrate mechanically and can be electrically sensed [17]. MEMS resonators attract interest in sensing applications, where changes in the frequency of resonant bodies are used to monitor certain quantities. In timing applications, a resonant plate can be connected to an electronic circuit to create a clock signal with excellent properties. It is also utilized in transmitters and receivers of radio frequency wireless devices [18]. 

MEMS resonators are demanded to be the main source of frequency generation in these applications due to low-cost batch fabrication [19]. Furthermore, it has the possibility for fabrication and integration with microelectronics at the die or package level [20]. These advantages lead to reduced cost and form-factor of the systems. Beyond all these advantages, there are still some limitations that remain unresolved. Some of these limitations are material loss, temperature instability [21], anchor loss, surface loss, ohmic loss, and capacitive loss. The main loss is anchoring loss, which is defined as the loss of energy due to the propagation of the acoustic wave from the resonant body to the supporting anchors. One way to save this energy is by adding an acoustic reflector [22]. The limitation of this technique Is the reflector is not efficient in reflecting acoustic waves [23]. Another way is to implement one or two-dimensional phononic crystals at the anchors of the resonator or in supporting tethers, which are widely performed by groups of researchers, and the difference in the effect of PnC structure to reflect acoustic wave by generating wide bandgap [24]. The third way is to mechanically isolate the vibration between the resonant body and anchor boundaries by adding a suspended frame between the anchors and the resonant body. Thakar et al. optimized the tether geometry (i.e., width and length) and PML scaling factor to obtain high anchor quality factor using (FEA) analysis method in COMSOL Multiphysics Software 5.5 for the lame-mode resonator, they found that the anchor loss is significant when the flexural-mode resonance frequency of tethers is synchronized with lame-mode resonator frequency [25]. Khine et al. applied two types of tethers in the lame-mode resonator (straight beam, T-shape beam) using perfect matched layer analysis in ABAQUS software; they found that adding a horizontal beam (T-shape beam) at tethers provides more freedom at the nodal point of the resonator and reduces the stress introduced by lateral rotation tilt of the resonance mode and, as a result, decreases loss and enhances the anchor quality factor [26]. Elsayed et al. optimized tether length and width in bulk-mode disk resonators by using a transverse piezoelectric actuation technique and fabricated by post-processing die process to reduce the width of the anchor beam supports and, as a result, reduces anchor loss [27]. In this work, firstly, the resonator dimensions such as the tether width, length and PML are designed for the highest achievable anchor quality factor with the conventional resonator structure using the finite element analysis method (FEA) in COMSOL Multiphysics. In this regard, the optimized dimensions with the lowest anchor loss are *tw* = 25 μm, *tl* = 1.5λ and PML dimensions of 3λ × 3λ. The conventional resonator structure with these optimized geometric values offers a *Q_anchor_* of 58,000. Then, a new tether design defined as a, tether with three legs (TWHL) is added to support anchors to minimize the transmitted force to anchors on the principle of force distribution/unit area. This structure helps to isolate the mechanical vibration between the resonant body and anchors, resulting in an enhanced stored energy in the resonant body so that the anchor quality factor increases. Stress distribution on the anchoring boundary is estimated using analytical calculation and the impact of the new design on the transmission characteristics is studied using the finite element simulation. With the deployment of the new tether structure, the *Q_anchor_* is enhanced to 175,000 which is 3-fold improvement compared to *Q_anchor_* of the conventional structure. 

## 2. Nonconventional Tethers Mechanism

**Figure 1:**
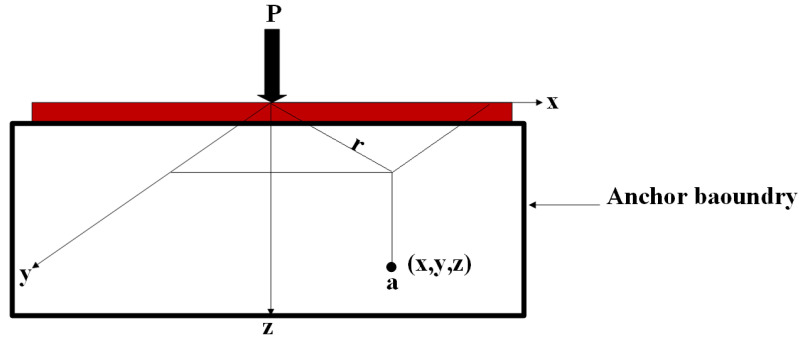
Illustration of stress at point (a) caused by point load *P* on the surface.

A new structure Tether with Three Legs (TWHL) is applied on the MEMS resonator support tethers. The concept of the new design is built on force distribution on the surface area. The force applied on anchor boundaries will be distributed along the three tether legs before reaching the anchors, resulting in a decrease in mechanical damping and quality factor enhancement. The mathematical relation for calculating the shear and normal stress inside elastic, isotropic, and homogenous mediums, due to the force applied at the surface, was developed by Boussinesq in 1885, as shown in Figure 1. According to this relationship, the stress at point (a) due to the force of magnitude *P* is given by [28]:

(1) $$\Delta \sigma =\frac{3P}{2\pi z^{2\;}{\left[1+{\left(\frac{r}{z}\right)}^{2}\right]}^{5/2}}$$

where Δ*σ* represents stress at point (a), *P* represents applied force $r=\sqrt{x^{2\;}+y^{2}}$, and *x*, *y*, *z* represents coordinates at point (a) as shown in Figure 1.

As shown in Figure 1 an infinite line load length has stress/unit length (*q*/*l*) on the surface of elastic material. The vertical stress, Δ*σ*, inside the surface at point (a), can be calculated using the theory of elasticity, or

(2) $$\Delta \sigma =\frac{{2qz}^{3}}{\pi {\left(x^{2}+z^{2}\right)}^{2}}$$

This equation can be rewritten as

(3) $$\Delta \sigma =\frac{2q}{\pi z{\left({\left(\frac{x}{z}\right)}^{2}+1\right)}^{2}}\;\;$$

(4) $$\frac{\Delta \sigma }{q/z}=\frac{2}{\pi {\left({\left(\frac{x}{z}\right)}^{2}+1\right)}^{2}}$$

Hence, Equation (4) is in a non-dimensional form. The calculation of the variation of Δ*σ*/(*q*/*z*) with *x*/*z* can be obtained using this equation. The value of Δ*σ* obtained from Equation (4) is the additional stress on the surface caused by the line load. To calculate the stress in conventional tether design at point (a) with coordinates *x* = 150 μm and *z* = 100 μm, lets substitute tether length (*l*) equal 75 μm in to Equation (3):

$$\Delta \sigma =\frac{2(\frac{q_{0}}{l})}{\pi \times 100{\left({\left(\frac{150}{100}\right)}^{2}+1\right)}^{2}}\;\;\;\;\;\;\;=\;8q_{0}\;$$

This shows that the stress increases inside the anchor boundary due to force transferred from tethers to a value equal to 8*q*_0_.

**Figure 2:**
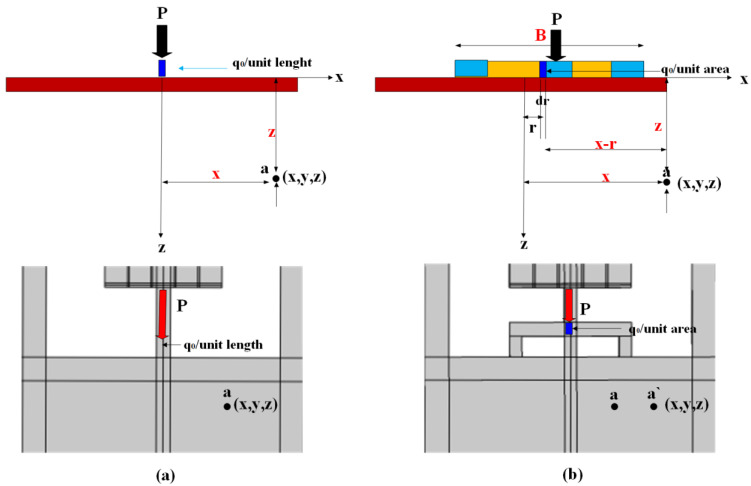
Illustration of (**a**) stress/unit length on surface and resonator (**b**) stress/unit area on surface and resonator.

Figure 2a represents the stress applied on anchor boundaries through the tether length (*q*/unit length). The stress applied in Figure 2b is distributed over the new design area (TWHL) if the design length is *l* and width is *B*, so the stress applied on the anchor is equal (*q*/BL), which is less than the stress on the line load (conventional tether). To prove the stress applied on anchors is lowered due to the TWHL tether, use Equation (2) to calculate the stress inside the surface width *B*. Assume a strip with force (*q*_0_ per unit area) as shown in Figure 2b. Consider an elemental strip of width *dr*, the force per unit length of this strip is equal to *q*_0_ *dr*. This elemental strip is considered a line load. Equation (4) gives the stress Δ*σ* at point (a) inside the surface caused by this elemental strip load. To determine the stress, substitute *q*_0_ dr for *q* and (*x* − *r*) for *x*. So,

(5) $$d\sigma =\frac{{2(q_{0}dr)z}^{3}}{\pi {\left({\left(x-r\right)}^{2}+z^{2}\right)}^{2}}\;\;$$

The stress Δ*σ* at point (a), caused by the given strip force of width *B*, can be calculated using the integration of Equation (5) with limits of r from −*B*/2 to + *B*/2, or

(6) $$\Delta \sigma =\int d\sigma =\int_{-B/2}^{+B/2}\left(\frac{2q}{\pi }\right)\left\{\frac{z^{3}}{{({\left(x-r\right)}^{2}+z^{2)}}^{2}}\right\}dr$$

(7) $$=\frac{q_{0}}{\pi }\left\{{tan}^{-1}\left[\frac{z}{x-\left(\frac{B}{2}\right)}\right]-{tan}^{-1}\left[\frac{z}{x+(\frac{B}{2})}\right]-\frac{Bz(x^{2}-z^{2}-(\frac{B^{2}}{4})}{{\left(x^{2}+z^{2}-\left(\frac{B^{2}}{4}\right)\right)}^{2}+B^{2}z^{2}}\right\}$$

Concerning Equation (6), the following should be considered:

${tan}^{-1}\left[\frac{z}{x-(\frac{B}{2})}\right]\;and\;{tan}^{-1}\left[\frac{z}{x+(\frac{B}{2})}\right]$ are in radiance, the magnitude of Δ*σ* depends on *x*/*z*, equation is valid if *x* ≥ *B*/2; however, for 0 ≤ *x* ≤ *B*/2 the magnitude of ${tan}^{-1}\left[\frac{z}{x-(\frac{B}{2})}\right]$ becomes negative. In this case, it should be replaced by ${\pi +tan}^{-1}\left[\frac{z}{x-(\frac{B}{2})}\right].$

To calculate the value of stress at point (a) due to applied load *P* on the resonator, in this work, *B* = 250 µm at point (a) *x* = 50 μm and *z* = 100 μm, and solve Equation (4) to obtain the value of stress. Since *x* = 50 < *B*/2 = 125 μm, and *q*_0_ represents stress/unit area applied on tether leg, Equation (6) can be rewritten as [28]:

(8) $$\Delta \sigma =\frac{q_{0}}{\pi }\left\{{tan}^{-1}\left[\frac{z}{x-\left(\frac{B}{2}\right)}\right]+\pi -{tan}^{-1}\left[\frac{z}{x+(\frac{B}{2})}\right]-\frac{Bz(x^{2}-z^{2}-(\frac{B^{2}}{4})}{{\left(x^{2}+z^{2}-\left(\frac{B^{2}}{4}\right)\right)}^{2}+B^{2}z^{2}}\right\}$$

Substitute in the equation

$${tan}^{-1}\left[\frac{z}{x-\left(\frac{B}{2}\right)}\right]={tan}^{-1}\left[\frac{100}{50-\left(\frac{250}{2}\right)}\right]={tan}^{-1}\left[-1.3\right]=-0.92$$

$${tan}^{-1}\left[\frac{z}{x+\left(\frac{B}{2}\right)}\right]={tan}^{-1}\left[\frac{100}{50+\left(\frac{250}{2}\right)}\right]={tan}^{-1}\left[0.57\right]=0.51$$

$$\frac{Bz(x^{2}-z^{2}-(\frac{B^{2}}{4})}{{\left(x^{2}+z^{2}-\left(\frac{B^{2}}{4}\right)\right)}^{2}+B^{2}z^{2}}=250*100\frac{\left[50^{2}-100^{2}-\left(\frac{250^{2}}{4}\right)\right]}{{\left[50^{2}+100^{2}-\left(\frac{250^{2}}{4}\right)\right]}^{2}+250^{2}*100^{2}}=-0.91$$

Hence, $\frac{\Delta \sigma }{q_{0}}=\frac{1}{\pi }\left[-0.92+\pi -0.51-(-0.91)\right]=0.83$, so $\Delta \sigma =0.83q_{0}$, the stress on point (a) lower than the applied stress on the tether legs by 17%. This verifies that the amount of stress force transferred to anchor boundaries was lowered due to the desired TWHL tether design. Similarly, at point (a) *x* = 150 > *B*/2 = 125 μm, substitute in Equation (6) as follows:

$$\Delta \sigma =\frac{q_{0}}{\pi }\left\{{tan}^{-1}\left[\frac{100}{150-\left(\frac{250}{2}\right)}\right]-{tan}^{-1}\left[\frac{100}{150+(\frac{250}{2})}\right]-\frac{250\times 100(100^{2}-100^{2}-(\frac{125^{2}}{4})}{{\left(150^{2}+100^{2}-\left(\frac{250^{2}}{4}\right)\right)}^{2}+125^{2}{\times 100}^{2}}\right\}$$

$${tan}^{-1}\left[\frac{z}{x-\left(\frac{B}{2}\right)}\right]={tan}^{-1}\left[\frac{100}{150-\left(\frac{250}{2}\right)}\right]={tan}^{-1}\left[4\right]=1.3$$

$${tan}^{-1}\left[\frac{z}{x+\left(\frac{B}{2}\right)}\right]={tan}^{-1}\left[\frac{100}{150+\left(\frac{250}{2}\right)}\right]={tan}^{-1}\left[0.36\right]=0.34$$

$$\frac{Bz(x^{2}-z^{2}-(\frac{B^{2}}{4})}{{\left(x^{2}+z^{2}-\left(\frac{B^{2}}{4}\right)\right)}^{2}+B^{2}z^{2}}=250\times 100\frac{\left[150^{2}-100^{2}-\left(\frac{250^{2}}{4}\right)\right]}{{\left[150^{2}+100^{2}-\left(\frac{250^{2}}{4}\right)\right]}^{2}+250^{2}\times 100^{2}}=-0.085$$

$\frac{\Delta \sigma }{q_{0}}=\frac{1}{\pi }\left[1.3-0.34-(-0.085)\right]=0.33$, so $\Delta \sigma =0.33q_{0}$, the stress on point (a) is lower than the applied stress on the tether legs by 67%. The two examine points in-and-out plan of the new tether design proved the ability of the new design to reduce the stress force transferred to anchors, resulting in energy stored enhancement.

## 3. Nonconventional Tethers Transmission Characteristics

**Figure 3:**
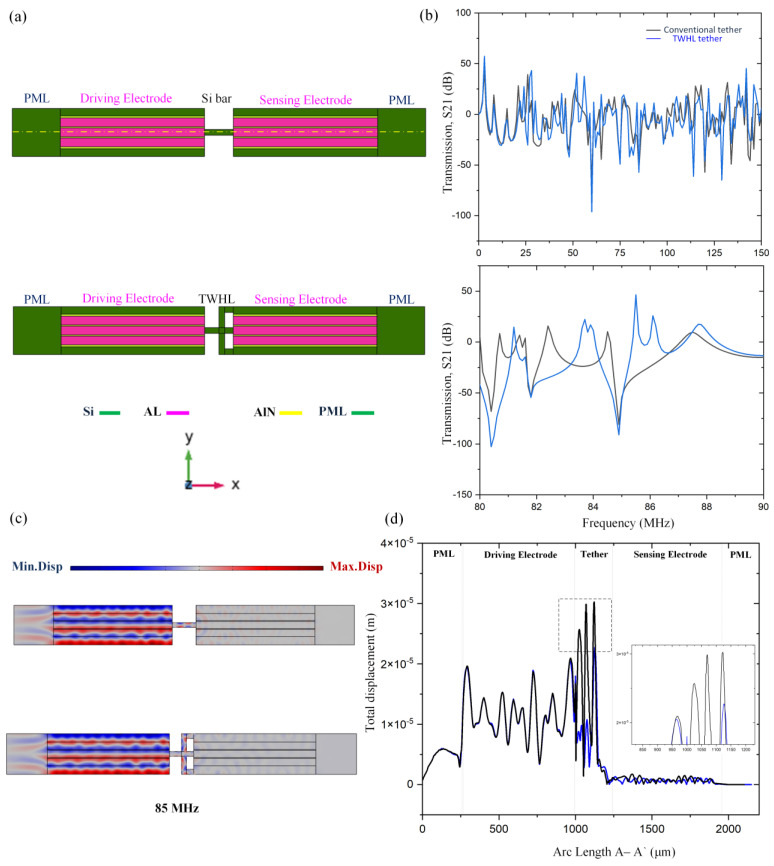
(**a**) 3D illustration of silicon bar and TWHL (**b**) transmission characteristics of silicon bar and TWHL (**c**) Displacement distribution of the delay line with silicon bar and TWHL as two transmission medium (**d**) displacement profile at 85 MHz frequency for the two delay lines on the A–A’.

To verify the acoustic wave energy transmission attenuation through the nonconventional tethers, two transmission lines were designed between the resonant body and anchor boundaries. The first one is a silicon bar and the second one is (TWHL), as shown in Figure 3. Figure 3b shows the TWHL structure has a lower transmission than the silicon bar, resulting in energy transfer attenuation. However, TWHL shows higher attenuation in the tether section compared to the conventional tether. The drive electrode is excited by 0.01 watts and sense electrodes are set to 0.0 watts. The transmission (S21) is calculated by [29,30,31,32,33]:

(9) $$S21\left(dB\right)=10{log}_{10}\left(\frac{P_{out}}{P_{in}}\right)\;$$

where P_in_ and P_out_ represent the amplitude of input and output power in the two transmission lines. The displacement distribution in the resonant body with two delay lines is shown in Figure 3c. S21 represents the input-to-output port power transmission coefficient. Clearly from Figure 3d at 85 MHz, the displacement profile for the delay line with TWHL shows strong attenuation to displacement than the silicon bar as they were used as two transmission mediums on the A–A’ line. The finite element simulation results prove that the wave is strongly attenuated in the transmission spectra at the beginning of the TWHL tether design and continues to zero, compared to the conventional tether. TWHL satisfies that there is strong prevention of propagation of acoustic waves.

## 4. Resonator Design

**Figure 4:**
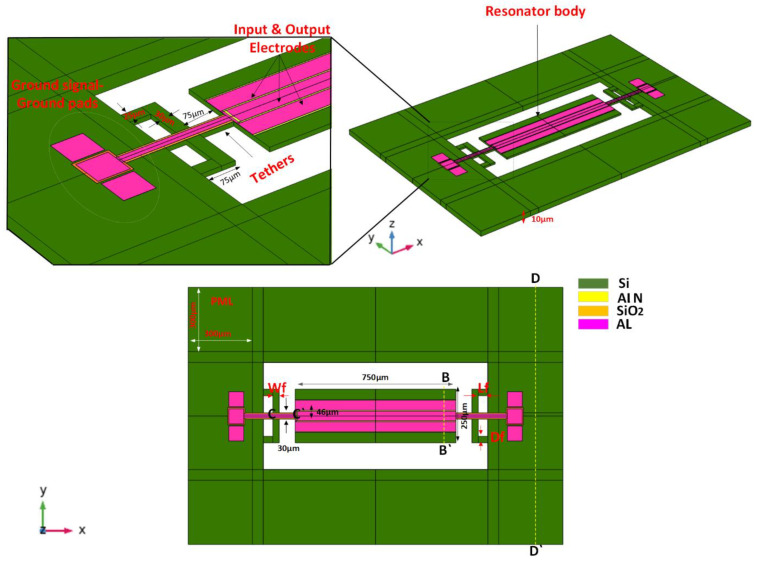
3D view of the resonator with TWHL tether structure.

A fifth-order width extension mode resonator is designed and simulated using COMSOL Multiphysics 5.5 through the finite element analysis method. As shown in Figure 4a, a thin layer (AlN) of 0.5 μm thickness, width, and length of 150 μm and 750 μm are placed on top of the silicon substrate. The dimensions of the resonator are 250 μm width and 750 μm length, respectively. The depth of the Al electrode is equal to 0.5 μm, width and length 46 μm and 750 μm, respectively, and the distance between the two electrode center lines is 50 μm (i.e., electrode gap = 4 μm). The electrode excites the vibration on the resonant body by applying 1V on the piezoelectric material. The wavelength of the resonator λ is equal to 100 μm. The dimensions of the TWHL are Lf = 45 μm, Df = 25 μm, and Wf = 30 μm as shown in Figure 4. The *f_r_* of the resonator can be calculated using the formula [34,35,36,37]:

(10) $$f_{r}=\frac{nv}{2w}$$

where *v* represents the velocity of the acoustic wave 8500 m/s, n represents the mode number equal to 5, and w represents the width of the resonator. The resonant frequency (*f_r_*) calculated from Equation (10) is ≈ 85 MHz in the desired design *w* = 250 μm. The resonator design parameters are illustrated in Table 1.

## 5. Resonator Mode Shapes

**Figure 5:**
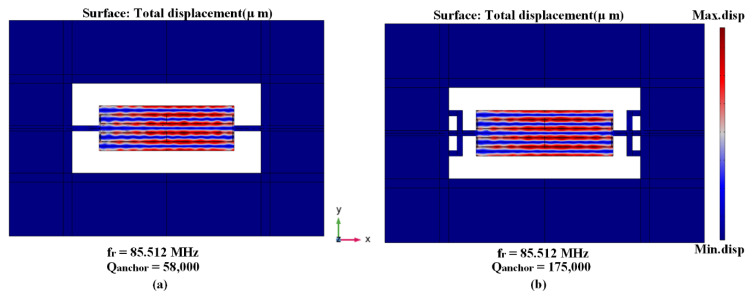
5th-order eigen mode shape and the calculated Q_anchor_ (**a**) with conventional tether and (**b**) with TWHL.

A Three-Leg Tether (TWHL) is applied in the tethers of the MEMS resonator to enhance the anchor quality factor and attenuate the energy leakage, and at the end enhance the total quality factor (*Q_tot_*). The *Q_anchor_* of the TPoS MEMS resonator can be obtained generally from the resonance frequency divided by the -3dB of bandwidth of the resonance maximum peak in the displacement profile at frequency response as [38,39,40,41]:

(11) $$Q_{anchor}=\frac{f_{r}}{∆f(-3\;dB)}$$

where *f_r_* is defined as resonance frequency, the anchor quality factor obtained from conventional tether is equal to 58,000, while the anchor quality factor of TWHL is equal to 175,000, providing a three-fold enhancement. The resonator mode shape with conventional tether and with (TWHL) and associated anchor quality factor are shown in Figure 5.

## 6. Discussion

**Figure 6:**
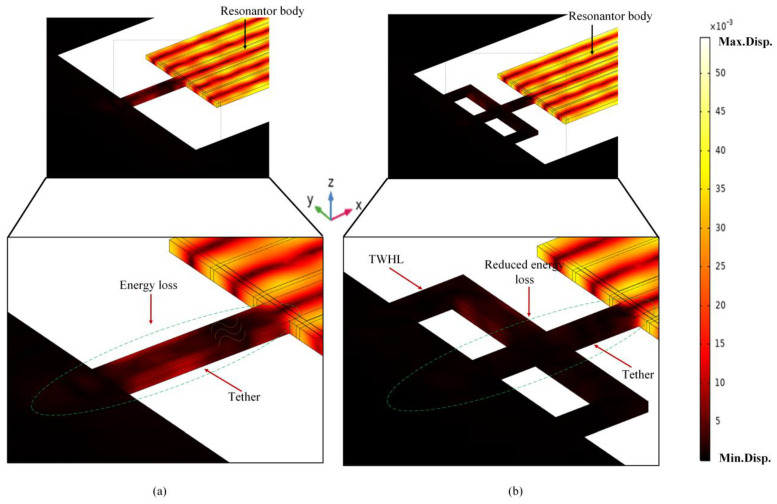
Illustration of leaked energy in (**a**) conventional tether and (**b**) TWHL tether.

The energy distribution of the bulk acoustic wave in conventional tether and TWHL is shown in Figure 6a,b. In the conventional tether, the amount of energy escaped from the resonant body is greater than the proposes design (see Figure 6a). The light brown color in the figure shows the energy transferred from the resonant body to the supporting anchors. Adding the TWHL tether decreases the amount of energy loss and increases the stored energy in the resonant body (see Figure 6b); the light brown color represents the amount of energy leaked to the supporting anchors, which is clearly less than the conventional tether. 

**Figure 7:**
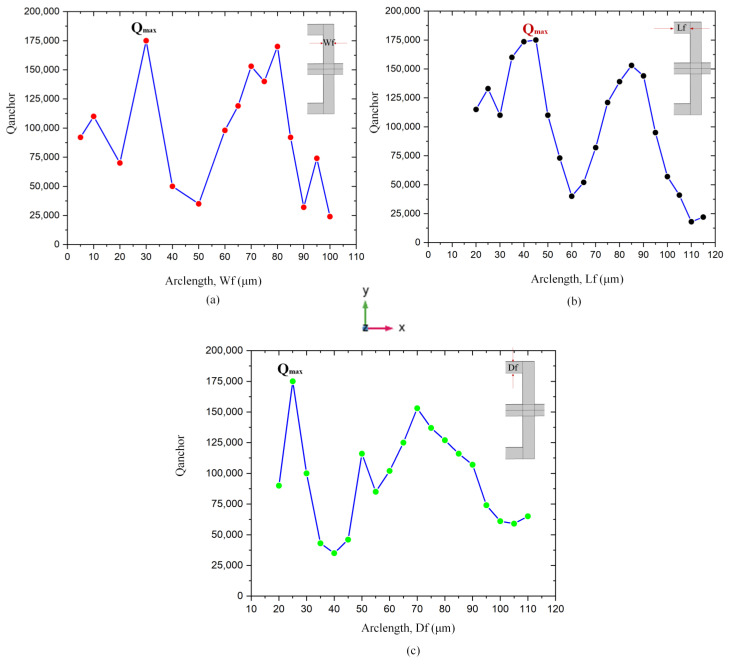
(**a**–**c**) Illustration of the effect of change TWHL dimensions on anchor quality factor.

Different values of anchor quality factor according to change TWHL dimensions (i.e., *Df*, *Wf*, *Lf*) is obtained as illustrated in Figure 7a–c. The branch *Wf* is swept from 5 μm to 100 μm, the maximum *Q_anchor_* obtained at width 30 μm. The length of TWHL tether in *x* direction is changed from 20 μm to 115 μm, the minimum value of the anchor quality factor is obtained at *Lf* = 110 μm, while the maximum *Q_anchor_* is at *Lf* = 45 μm. The depth of the TWHL in the y direction is swept from 20 μm to 110 μm, while *Wf*, *Lf* is set to 30 μm and 45 μm, respectively. The maximum *Q_anchor_* at the values of *Df* = 25 μm. All the curves show the periodic change with design geometry with maximum and minimum points.

**Figure 8:**
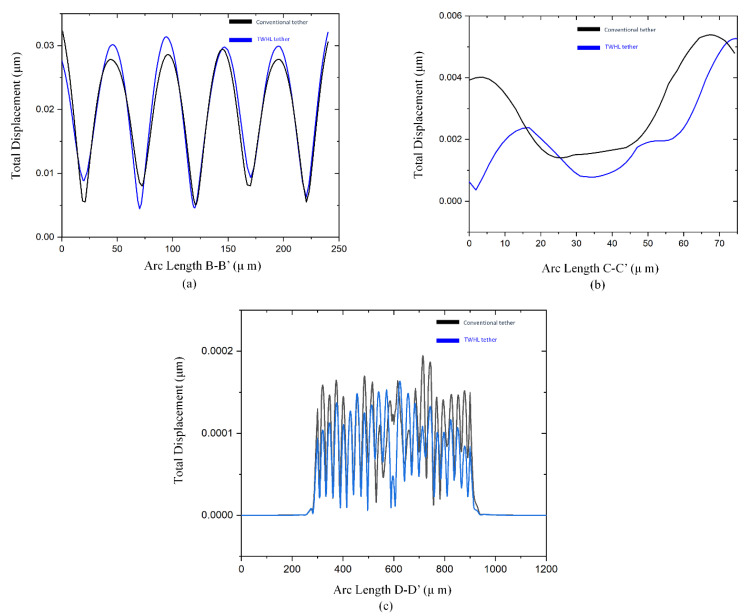
Total displacement fields of two resonators conventional and TWHL across the lines (**a**) B–B’, (**b**) C–C’, and (**c**) D–D’.

To prove the attenuation of the acoustic wave’s propagation through the supporting tethers, two designs of resonators were applied. The first resonator is a conventional tether, the second resonator is a TWHL resonator. The displacement profile of the resonant body at line B-B’ in Figure 8a shows that the resonator with TWHL has a total displacement of a resonant body equal to 0.32 μm, which is the highest displacement. This confirms that the energy stored in the TWHL resonator is higher than the conventional resonators. On the other hand, Figure 8b, c at the lines C-C’ and D-D’ (see Figure 4) verify that the resonator with TWHL has lower displacements at the tethers and anchoring boundaries, resulting in minimum energy leakage. This confirms the amount of energy stored in the resonant body of the resonator with TWHL is higher due to the prevention of energy escape to the anchors. As a result, a *Q_anchor_* of value equal to 17,500 was achieved from the TWHL resonator compared to a *Q_anchor_* of value equal to 58,000 from the conventional resonator, resulting in three-fold improvements. 

**Figure 9:**
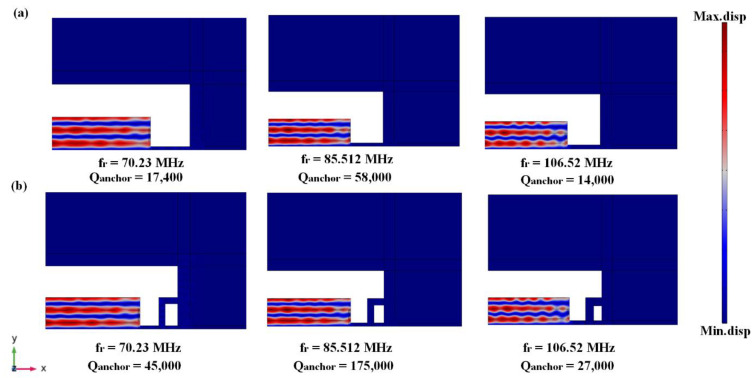
Quarter eigenmode shapes and associated anchor quality factor under different resonance frequencies (**a**) with conventional tether and (**b**) with TWHL.

Figure 9 illustrates the conventional and TWHL resonator vibration modes with different resonance frequencies 70.23 MHz, 85.51 MHz (desired design), and 106.5 MHz. The three resonators operate in the fifth order vibration mode, while the maximum anchor quality factor appears at a resonator operation of 85.5 MHz with a conventional tether and TWHL, which achieves minimum energy loss.

**Figure 10:**
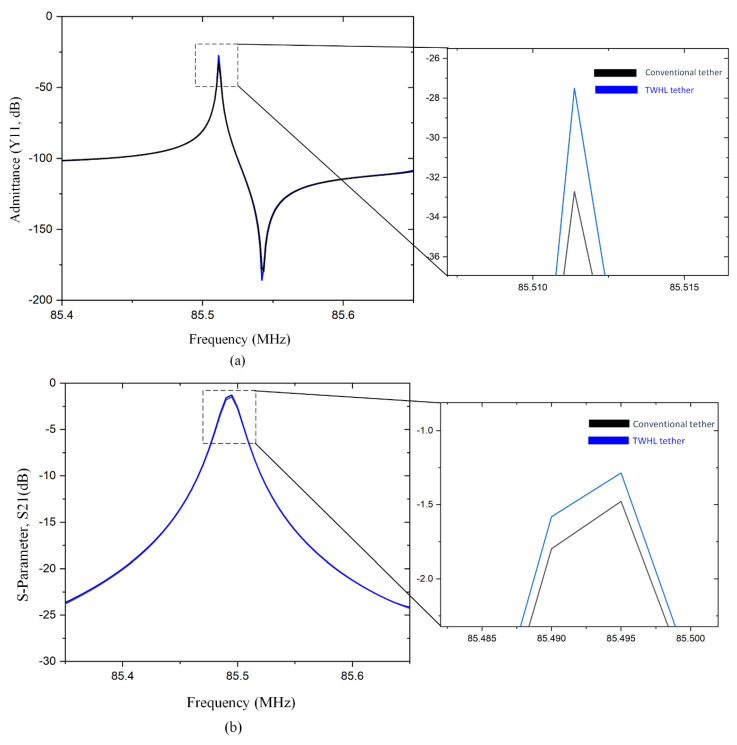
Simulated (**a**) admittance, (Y11) plot of the conventional and TWHL resonators (**b**) S21 (dB) characterizations plot of the conventional and TWHL resonators.

The resonator performance characteristics admittance Y_11_ and S_21_ (dB) of the two resonator designs (TWHL, conventional) are illustrated in Figure 10a,b. The summaries of the results are k^2^_eff_ of 0.08% is obtained from the TWHL resonator. Continuously, the simulated parameters such as insertion loss (*IL*) are 1.25 dB, the loaded quality factor (*Q_l_*) is 3842, and the unloaded quality factor (*Q_u_*) can be calculated by [29]:

(12) $$Q_{u}=\frac{Q_{l}}{1-10^{\left(\frac{-IL}{20}\right)}}$$

where the *Q_l_*-loaded quality factor in this design is 3842, (*IL*) is the resonator insertion loss equal to 1.25, (*Q_u_*) obtained from Equation (12) is equal to 27,442, which accounts for an almost 1.2-fold improvement, as illustrated in Table 2.

## 7. Conclusions

In this work, a new tether design was applied to a thin-film piezoelectric-on-Si micromachined resonator to enhance the anchor quality factor. The design and simulated results show significant enhancement in anchor quality factor from 58,000 to 175,000, representing three-fold improvements compared to a conventional tether design resonator. Continuously, the obtained *Q_u_* of the resonator with TWHL was 27,442 at 85 MHz, achieving a 1.2-fold enhancement in unloaded quality factor in comparison with a conventional resonator. The insertion loss was lowered from 1.5 dB to 1.25 dB for the proposed resonator design. The TWHL resonator performance parameters are significantly better than the conventional resonator. 

## Figures and Tables

**Table 1:** Resonator design parameters.

Parameter	Value (μm)
Length of resonator, *l*	750
Width of resonator, *W*	250
Thickness of piezoelectric layer, *Pt*	0.5
Thickness of electrode, *Ew*	0.5
Length of tether, *tl*	1.5λ
Width of tether, *tw*	12
Electrode thickness	0.4
Resonant frequency, *fr*	85 (MHz)
Wavelength, *λ*	100
Electrode gap	4
Silicon substrate high	10
Perfect matched layer width	3λ
TWHL depth, *Df*	25
TWHL length, *Lf*	45
TWHL width, *Wf*	30

**Table 2:** Performance of the TWHL resonator and conventional resonator.

Resonator	*fr* (MHz)	*Q_anchor_*	*IL* (dB)	*Q_u_*	K^2^_eff_%	*R_m_* (Ω)	FoM
T-Shape Tether [42]	10	87,449	22.26	4522	0.09	-	4
PnC Strip Tethers [43]	109	1,876,000	32	9744	0.035	3468	0.34
Conventional	85.5	58,000	1.5	23,750	0.08	10	19
With TWHL	85.5	175,000	1.25	27,442	0.08	9.4	22

## Data Availability

Not applicable.
